# Prediction and analysis of natural gas consumption in chongqing with a grey prediction model group in the context of COVID‐19

**DOI:** 10.1002/ese3.1164

**Published:** 2022-04-28

**Authors:** Bo Zeng, Shuangyi Yang, Cuiwei Mao, Dehai Zhang

**Affiliations:** ^1^ School of Management Science and Engineering Chongqing Technology and Business University Chongqing China; ^2^ College of Wealth Management Chongqing Finance and Economics College Chongqing China

**Keywords:** Chongqing's natural gas consumption, COVID‐19, grey prediction model group, prediction and analysis, random oscillation sequence

## Abstract

In this paper, a grey prediction model group is employed to quantitatively study the impact of COVID‐19 on natural gas consumption in Chongqing, China. First, a grey prediction model group suitable for the prediction of Chongqing's natural gas consumption is introduced, which consists of GM(1,1), TWGM(1,1), and the newly‐developed ODGM(1,1). Then, the model group is constructed to predict Chongqing's natural gas consumption in 2020. Finally, compare the predicted results of the model group with the actual consumption and quantitatively analyze the impact of the epidemic on natural gas in Chongqing. It is found that the impact of the epidemic on the consumption of natural gas in the first quarter of the year is very small, but relatively bigger in the second and third quarters. The study is of positive significance to maintain the supply and demand balance of natural gas consumption in Chongqing in the background of COVID‐19; and it enriches and develops the theoretical system of grey prediction models.

## Graphical abstract

A grey prediction model group is employed to quantitatively study the impact of COVID‐19 on natural gas consumption in Chongqing, China. The result shows that the impact of the epidemic on the consumption of natural gas in the first quarter of 2020 is very small, but relatively bigger in the second and third quarters.

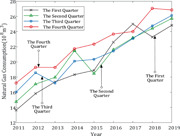

## 1 INTRODUCTION

A highly contagious novel coronavirus disease (COVID‐19) has swept the globe in early 2020 and caused tremendous changes in normal social life. Industry, transportation, commerce, and other industries closely related to natural gas consumption^1^ have been greatly affected. As one of the largest natural gas consumption cities in China, Chongqing has abundant natural gas resources and numerous natural gas transportation networks. According to statistics, the proportion of natural gas consumption in the total energy consumption in Chongqing is higher than the national average.^2^ COVID‐19 will probably continue for a long time. Hence, it is of positive significance to study the impact of COVID‐19 on the change of natural gas consumption in Chongqing to ensure the balance of natural gas supply and demand in Chongqing.

A stable supply of natural gas is necessary for people's happy life and the sustainable development of the society. However, the consumption of natural gas in Chongqing has also undergone an abnormal change under the influence of COVID‐19, making the previous consumption data less reliable when used as references in the supply of natural gas. Since it is important for Chongqing's government to formulate effective energy policies and ensure the stable supply of natural gas, scientifically forecasting the consumption of natural gas and analyzing the reasons of the difference between the actual consumption and the predicted consumption in 2020 will serve as a reference in relative decision‐making.

Several methods have been used to predict natural gas consumption, such as time‐series method,^3^, ^4^, ^5^ neural network,^6^, ^7^ and econometric model.^8^ When there are enough data, these models can predict the natural gas consumption accurately. However, reliability of these models cannot be guaranteed when the sample size is small and the data contain great uncertainty. The data of natural gas consumption in Chongqing are just of this kind and bear the characteristics of small sample size and poor information. The reasons are possibly as follows: first, the energy consumption structure in Chongqing has greatly changed since 2011 due to the municipal government's initiative in developing low‐carbon energy for the sake of environmental conservation. This change makes the historical data of natural gas consumption in Chongqing before 2011 of little value in predicting the natural gas consumption in the future and means the data available for natural gas consumption forecasting are really limited; second, the emergence of COVID‐19 adds a lot of uncertainties to the circumstances.

Grey prediction models have been widely used and proved effective in dealing with research objects with small data, poor information, and great uncertainties. Fine examples can be easily found in energy,^9^, ^10^, ^11^, ^12^, ^13^ environment,^14^, ^15^ agriculture,^16^, ^17^ and other fields.^18^, ^19^, ^20^ In the system of grey prediction theory, GM(1,1) is the most primitive and classical model. It is suitable for data sequences with approximately homogeneous exponential growth. However, when data sequences are more complicated, the simulation errors of GM(1,1) will be large.^21^, ^22^ To solve the problem, a series of studies have been conducted and fruitful research findings have been obtained. Zeng et al.^23^ proposed a new‐structure grey Verhulst model which improved the modeling ability of grey model for saturated S‐shaped sequences. Based on the traditional GM(1,1) modeling mechanism, Zhan and Shi^24^ proposed a grey model fit for nonhomogenous exponential sequences and gave out the model parameters with least square solutions as well as its time response function. Zeng et al.^25^ proposed a new multivariable grey prediction model which can be completely compatible with the mainstream single variable and multivariable grey prediction models by adjusting and changing the model's parameters.

The studies above had improved the structures of grey prediction models gradually and expanded the scope of application of grey prediction models. Nevertheless, all the above‐mentioned models could not ensure the reliability of prediction when faced with data sequences with poor overall smoothness. For example, for the random oscillation sequences widely existing in the real world, the simple data accumulation processing cannot improve its smoothness significantly and the accuracy of prediction models is difficult to achieve.

In this paper, a new grey prediction model (ODGM(1,1)) for the prediction of natural gas consumption in Chongqing in the fourth quarter of 2020 was proposed. This model can increase the simulation and prediction accuracy of overall oscillation sequences and is fit for the prediction for nonlinear systems with overall oscillation.

In the following sections, different abbreviations for various grey prediction models will be used and the specific abbreviations and their definitions are listed in Table 1.

**Table 1:** Abbreviations and definitions for various grey prediction models

No.	Abbreviation	Definition
1	GM(1,1)^26^	Grey model with one variable and one first‐order equation
2	TWGM(1,1)^22^	Three‐parameter whitenization grey model
3	TDGM(1,1)^22^	Three‐parameter discrete grey model
4	ODGM(1,1)	Oscillatory and discrete grey model with one variable and one first‐order equation
5	NGM(1,1,*k*)^27^	Nonhomogeneous grey model
6	DGM(1,1)^28^	Discrete grey model with one variable and one first‐order equation

The reminder of this paper is organized as follows. In Section 2, the data characteristics of quarterly natural gas consumption in Chongqing is analyzed and the common forms of GM(1,1) and TWGM(1,1) are defined. In Section 3, a new grey prediction model ODGM(1,1) is proposed and its time response equation is deduced. In Section 4, a grey prediction model group is constructed, consisting of GM(1,1), TWGM(1,1) and the newly‐developed ODGM(1,1), to simulate and predict the quarterly natural gas consumption of Chongqing from 2011 to 2020, compare the prediction value with the actual value in 2020 and analyze the impact of COVID‐19 on the natural gas consumption in Chongqing. In Section 5, conclusions are summarized.

**Figure 1:**
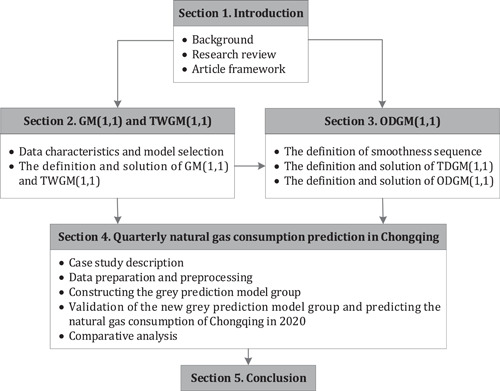
Structure of this paper.

To clearly represent the structural relationship between various parts, the structure chart of the paper is given as Figure 1.

## 2 GREY SYSTEM PREDICTION MODEL

### 2.1 Data characteristics and model selection

**Figure 2:**
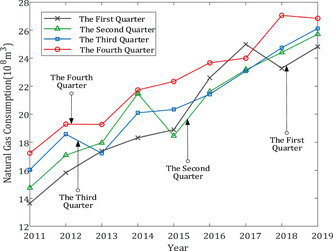
Natural gas consumption in Chongqing from 2011 to 2019

The quarterly consumption of natural gas in Chongqing from 2011 to 2019 showed a general upward trend but bore random oscillation. However, although consumption in the first three quarters of some years, as is shown in Figure 2, was sort of oscillatory, the sequences were generally smooth and showed the characteristics of approximate nonhomogeneous exponential growth. After accumulation processing, the smoothness of the first three quarter sequences has been improved. However, the fourth quarter sequence showed the characteristics of global oscillation, as is shown in Figure 1. Thus, we improve the smoothness of the fourth quarter sequence before modeling to make sure the simulation and prediction accuracy be higher than that by direct modeling. Therefore, a novel grey prediction model called ODGM(1,1) based on smooth operator will be established to predict natural gas consumption of Chongqing in the fourth quarter of 2020.

Grey prediction theory based on GM(1,1) has matured after decades of development.^29^, ^30^, ^31^, ^32^ The time response equation of GM(1,1) is a homogeneous exponential function, so GM(1,1) has a good simulation effect for homogeneous exponential sequence and approximate homogeneous exponential sequence.^22^ TWGM(1,1) is one of the most widely‐used models in the field of grey prediction theory. It expresses the relationship between grey actions and the time point and adds the random disturbance term to weaken the effects of other information or factors except for the main factor. Meanwhile, the time response equation of TWGM(1,1) is a homogeneous exponential function, so TWGM(1,1) has a good simulation effect for nonhomogeneous exponential sequence and approximate nonhomogeneous exponential sequence.^22^ Therefore, fully considering the data characteristics, development trend of quarterly natural gas consumption in Chongqing from 2011 to 2019 and structural characteristic of models, TWGM(1,1) is used to simulate and predict the natural gas consumption in the first and third quarters, GM(1,1) to simulate and predict the natural gas consumption in the second quarter and ODGM(1,1) to simulate and predict the natural gas consumption in the fourth quarter.

### 2.2 GM(1,1) and TWGM(1,1)

Definition 1 ((Liu and Lin^21^)) Assume that the original sequence is $x^{\left(0\right)}\left(k\right)\geq 0,k=1,2,\text{⋯},n$. Then $X^{\left(1\right)}=\left(x^{\left(1\right)}\left(1\right),x^{\left(1\right)}\left(2\right),\text{⋯},x^{\left(1\right)}\left(n\right)\right)$ is the 1‐Accumulating Generation Operator (1‐AGO) sequence of $X^{\left(0\right)}$ and $Z^{\left(1\right)}=\left(z^{\left(1\right)}\left(2\right),z^{\left(1\right)}\left(3\right),\text{⋯},z^{\left(1\right)}\left(n\right)\right)$ is the mean sequence generated by consecutive neighbors of $X^{\left(1\right)}$, where

$$x^{\left(1\right)}\left(k\right)=\sum_{i=1}^{k}x^{\left(0\right)}\left(k\right),k=1,2,\text{⋯},n$$

$$Z^{\left(1\right)}\left(k\right)=0.5\times \left[x^{\left(1\right)}\left(k\right)+x^{\left(1\right)}\left(k-1\right)\right],k=2,3,\text{⋯},n.$$

Definition 2 Assume *X* ^(0)^ and *X* ^(1)^ are stated as Definition 1, and then

(1) $$x^{\left(0\right)}\left(k\right)+az^{\left(1\right)}\left(k\right)=b$$

is the basic form of GM(1,1).

Theorem 1 ((Zeng et al.^22^)) Assume GM(1,1) is stated as Definition 2, and then its time response equation is as follows:

(2) $${\hat{y}}_{k+1}=\left(1-e^{a}\right)\left(y_{1}-\frac{b}{a}\right)e^{-ak},k=1,2,\text{⋯},n-1,\text{⋯}$$

where

$${\left(a,b\right)}^{T}={\left(B^{T}B\right)}^{-1}B^{T}A,A=\left(\begin{array}{l} y\left(2\right)\\ y\left(3\right)\\ \text{⋮}\\ y\left(n\right) \end{array}\right),B=\left(\begin{array}{ll} -z_{1}^{\left(1\right)}\left(2\right) & 1\\ -z_{1}^{\left(1\right)}\left(3\right) & 1\\ \text{⋮} & \text{⋮}\\ -z_{1}^{\left(1\right)}\left(n\right) & 1 \end{array}\right).$$

Definition 3 Assume *X* ^(0)^ and *X* ^(1)^ are stated as Definition 1, and then

(3) $$x^{\left(0\right)}\left(k\right)+az^{\left(1\right)}\left(k\right)=kb+c$$

is three‐parameter whitenization grey model (TWGM(1,1)).^22^ The first‐order differential equation

(4) $$\frac{dx^{\left(1\right)}}{dt}+ax^{\left(1\right)}=bt+c$$

is called whitenization differential equation of TWGM(1,1).

Theorem 2 ((Zeng et al.^22^)) Assume $X^{\left(0\right)}$, $X^{\left(1\right)}$, and $Z^{\left(1\right)}$ are stated as Definition 1, and in Definition 3 specifically, the least square estimation of the sequence parameters $\hat{g}={\left(a,b,c\right)}^{T}$ of TWGM(1,1) satisfy:

(5) $$\hat{g}={\left(a,b,c\right)}^{T}={\left(B^{T}B\right)}^{-1}B^{T}A$$

where

$$A=\left(\begin{array}{l} x^{\left(0\right)}\left(2\right)\\ x^{\left(0\right)}\left(3\right)\\ \text{⋮}\\ x^{\left(0\right)}\left(n\right) \end{array}\right),B=\left(\begin{array}{lll} -z^{\left(1\right)}\left(2\right) & 2 & 1\\ -z^{\left(1\right)}\left(3\right) & 3 & 1\\ \text{⋮} & \text{⋮} & \text{⋮}\\ -z^{\left(1\right)}\left(n\right) & n & 1 \end{array}\right)$$

Theorem 2 can be proved by using the least square method.

The time response equation of TWGM(1,1) can be deduced by solving the differential Equation (4), as is described in Theorem 2.

Theorem 3 ((Zeng et al.^22^)) Assume TWGM(1,1) is stated as Definition 3, and then its time response equation is as follows:

(6) $${\hat{x}}^{\left(0\right)}\left(k\right)=\alpha e^{-a\left(k-1\right)}+\beta ,k=2,3,\text{…},n$$

where

$$\alpha =\left(1-e^{a}\right)\left(x^{\left(0\right)}\left(1\right)-\frac{b}{a}+\frac{b}{a^{2}}-\frac{c}{a}\right);\beta =\frac{b}{a}.$$

The detailed deduction process can be found in the study by Zeng et al.^22^

## 3 OSCILLATORY AND DISCRETE GREY MODEL

Different models are suitable for data sequences with different characteristics. For Chongqing's natural gas consumption in the fourth quarter since 2011, ODGM(1,1) performs better than other grey prediction models in simulation and prediction accuracy. Hence, we build ODGM(1,1) for the fourth‐quarter data sequence to predict the natural gas consumption in Chongqing if there were no epidemic in 2020.

Definition 4 Assuming that a raw sequence X=(x(1),x(2),…,x(n)) satisfies, when ∀k=2,3,…,n:

1. If x(k)−x(k−1)>0, the sequence X is a monotonically increasing sequence;
2. If x(k)−x(k−1)<0, the sequence X is a monotonically decreasing sequence;
3. If ∃k,k′∈{2,3,…,n} and x(k)−x(k−1)>0, x(k′)−x(k′−1)<0, the sequence X is called a random oscillation sequence.^32^

Assume *M* and *m* satisfy:

M=max{x(k)∣k=1,2,…,n};m=min{x(k)∣k=1,2,…,n}

and then T=M−m is the amplitude of the random oscillation sequence.

Definition 5 Assume the random oscillation sequence is X, Tis the amplitude of X and D is an operator acting on X. Let

XD=(x(1)d,x(2)d,…,x(n−1)d)

where

(7) $$x\left(k\right)d=\frac{\left[x\left(k\right)+T\right]+\left[x\left(k+1\right)+T\right]}{4},k=1,2,\text{…},n-1.$$

Then, D is called the first order smoothness operator of X, and XD is called the smoothness sequence of X.^32^

Definition 6 Assume $X^{\left(0\right)}$ and $X^{\left(1\right)}$ are stated as Definition 1, and then

(8) $$x^{\left(0\right)}\left(k\right)+az^{\left(1\right)}\left(k\right)=kb+c$$

is three‐parameter discrete grey model (TDGM(1,1)).^22^

The parameter estimation method of TDGM(1,1) is the same as that of TWGM(1,1). See Theorem 2 for details.

Whitenization differential equation of TWGM(1,1) is established, and then deduced functional relationship of TWGM(1,1) between ${\hat{X}}^{\left(0\right)}\left(k\right)$ and *k* by solving the differential equation. However, relationship of TDGM(1,1) between ${\hat{X}}^{\left(0\right)}\left(k\right)$ and *k* based on Equation (8) can be directly deduced. The time response equation of TDGM(1,1) is described in Theorem 4.

Theorem 4 ((Zeng et al.^22^)) Assume TDGM(1,1) is stated as Definition 6, and then its time response equation is as follows:

(9) $${\hat{x}}^{\left(0\right)}\left(k\right)=\alpha \beta^{k-2}+\sum_{g=0}^{k-3}\gamma \beta^{g},k=2,3,\text{⋯},n$$

where

$$\alpha =x^{\left(0\right)}\left(1\right)\left(\frac{1-0.5a}{1+0.5a}-1\right)+\left(2\times \frac{b}{1+0.5a}+\frac{c}{1+0.5a}\right);\beta =\frac{1-0.5a}{1+0.5a};\gamma =\frac{b}{1+0.5a}$$

The detailed deduction process can be found in the study by Zeng et al.^22^

Definition 7 Assume the random oscillation sequence is X, and the smoothness sequence Y of X and the mean sequence generated of $Z^{\left(1\right)}$ are as stated in Definition 5 and Definition 1, respectively, where Y=(y(1),y(2),…,y(n)). Then, the following grey equation

(10) $$y\left(k\right)+az^{\left(1\right)}\left(k\right)=kb+c$$

is called the basic form of ODGM(1,1).

Theorem 5 Assume ODGM(1,1) is stated as Definition 7, and its time response equation is as follows:

1. When t is an even number,

(11) $$\hat{x}\left(t+1\right)=\frac{4\alpha \left(1+\beta^{t-1}\right)}{1+\beta }+\frac{4\gamma \left(\beta -\beta^{t-1}\right)}{1-\beta^{2}}-x\left(2\right)-2T$$

2. When t is an odd number,

(12) $$\hat{x}\left(t+1\right)=\frac{4\alpha \left(\beta^{t-1}-1\right)}{1+\beta }+\frac{4\gamma \left(1-\beta^{t-1}\right)}{1-\beta^{2}}+x\left(2\right)$$

According to Equation (5), we can obtain:

$$\hat{x}\left(k+1\right)=4\hat{y}\left(k\right)-\hat{x}\left(k\right)-2T.$$

When k=1,

$$\hat{x}\left(2\right)=4\hat{y}\left(1\right)-\hat{x}\left(1\right)-2T=x\left(2\right),$$

x(2) is called the initial condition for building the new grey prediction model, and regarded as known data.

When k=2,

$$\hat{x}\left(3\right)=4\hat{y}\left(2\right)-x\left(2\right)-2T.$$

When k=3,

$$\hat{x}\left(4\right)=4\hat{y}\left(3\right)-\left(4\hat{y}\left(2\right)-x\left(2\right)-2T\right)-2T=4\left[\hat{y}\left(3\right)-\hat{y}\left(2\right)\right]+x\left(2\right).$$

When k=t,

(13) $$\hat{x}\left(t+1\right)=4\left[\hat{y}\left(t\right)-\hat{y}\left(t-1\right)+\text{…}+{\left(-1\right)}^{t}\hat{y}\left(2\right)\right]+{\left(-1\right)}^{t+1}x\left(2\right)-\left(1+{\left(-1\right)}^{t}\right)T.$$

Substituting Equation (9) into Equation (13) and rearranging it, we can obtain:

(14) $$\hat{x}\left(t+1\right)=4\alpha \left(\beta^{t-2}-\beta^{t-3}+\text{⋯}+{\left(-1\right)}^{t}\beta^{0}\right)+4\gamma \left(\sum_{g=0}^{t-3}\beta^{g}-\sum_{g=0}^{t-4}\beta^{g}+\text{⋯}+{\left(-1\right)}^{t}\sum_{g=0}^{0}\beta^{g}\right)+{\left(-1\right)}^{t+1}x\left(2\right)-\left(1+{\left(-1\right)}^{t}\right)T.$$

When t is an even number,

(15) $$\hat{x}\left(t+1\right)=4\alpha \left(\beta^{t-2}-\beta^{t-3}+\text{⋯}+\beta^{0}\right)+4\gamma \left(\beta^{t-3}+\beta^{t-5}+\text{⋯}+\beta \right)\;-x\left(2\right)-2T.$$

From Equation (15), it is easy to note that both $\left(\beta^{t-2}-\beta^{t-3}+\text{⋯}+\beta^{0}\right)$ and $\left(\beta^{t-3}+\beta^{t-5}+\text{⋯}+\beta \right)$ are equal ratio sequences, and their radios are as follows:

$$q_{1}=-\frac{\beta^{t-2}}{\beta^{t-3}}=-\frac{\beta^{t-3}}{\beta^{t-4}}=\text{⋯}=-\frac{\beta }{1}=-\beta ,$$

$$q_{2}=\frac{\beta^{t-3}}{\beta^{t-5}}=\frac{\beta^{t-5}}{\beta^{t-7}}=\text{⋯}=\frac{\beta^{3}}{\beta }=\beta^{2}.$$

According to the summation formula of equal ratio sequences, we can deduce that

(16) $$\hat{x}\left(t+1\right)=\frac{4\alpha \left(1+\beta^{t-1}\right)}{1+\beta }+\frac{4\gamma \left(\beta -\beta^{t-1}\right)}{1-\beta^{2}}\;-x\left(2\right)-2T.$$

When t is an odd number,

(17) $$\hat{x}\left(t+1\right)=4\alpha \left(\beta^{t-2}-\beta^{t-3}+\text{⋯}-\beta^{0}\right)+4\gamma \left(\beta^{t-3}+\beta^{t-5}+\text{⋯}+\beta^{2}+1\right)+x\left(2\right).$$

From Equation (17), it is easy to note that both $\left(\beta^{t-2}-\beta^{t-3}+\text{⋯}-\beta^{0}\right)$ and $\left(\beta^{t-3}+\beta^{t-5}+\text{⋯}+\beta^{2}+1\right)$ are equal ratio sequences, and their ratio are as follows:

$$q_{1}^{\prime }=-\frac{\beta^{t-2}}{\beta^{t-3}}=-\frac{\beta^{t-3}}{\beta^{t-4}}=\text{⋯}=-\frac{\beta }{1}=-\beta ,$$

$$q_{2}^{\prime }=\frac{\beta^{t-3}}{\beta^{t-5}}=\frac{\beta^{t-5}}{\beta^{t-7}}=\text{⋯}=\frac{\beta^{2}}{1}=\beta^{2}.$$

According to the summation formula of equal ratio sequence, we can deduce that

(18) $$\hat{x}\left(t+1\right)=\frac{4\alpha \left(\beta^{t-1}-1\right)}{1+\beta }+\frac{4\gamma \left(1-\beta^{t-1}\right)}{1-\beta^{2}}+x\left(2\right).$$

In summary, the time response equation of ODGM(1,1) is as follows: 1. When t is an even number,

(19) $$\hat{x}\left(t+1\right)=\frac{4\alpha \left(1+\beta^{t-1}\right)}{1+\beta }+\frac{4\gamma \left(\beta -\beta^{t-1}\right)}{1-\beta^{2}}\;-x\left(2\right)-2T.$$

2. Whentis an odd number,

(20) $$\hat{x}\left(t+1\right)=\frac{4\alpha \left(\beta^{t-1}-1\right)}{1+\beta }+\frac{4\gamma \left(1-\beta^{t-1}\right)}{1-\beta^{2}}+x\left(2\right).$$

## 4 QUARTERLY NATURAL GAS CONSUMPTION PREDICTION IN CHONGQING

### 4.1 Case study description

Energy is a necessary material for economic development. At present, China's energy structure is transforming to low‐carbon and green. In 2010, Chongqing was listed as a pilot city for the development and construction of a “low‐carbon city” by the National Development and Reform Commission of China. To this end, the Chongqing Municipal People's Government has adjusted its industrial structure, transformed its economic development mode, and actively promoted energy conservation and emission reduction. Natural gas is a green, clean and environmentally friendly primary energy. Compared with other traditional primary energy such as coal and oil, natural gas emits far less greenhouse gases in producing the same heat. Therefore, low‐carbon natural gas is a good transitional energy on the premise that it takes a long time to realize deep electrification and high permeability green hydrogen. Chongqing is one of the most mature regions in China's natural gas market. The proportion of natural gas consumption in total energy consumption in Chongqing is increasing year by year. In the primary energy consumption structure of Chongqing in 2019, natural gas accounted for more than 17%, far exceeding China's average level of 8% in the same year.^33^, ^34^ In Chongqing's gross domestic product (GDP), the production value of industries related to natural gas consumption such as industry, transportation, postal service and storage accounts for a considerable part of Chongqing's GDP.^33^, ^34^ Therefore, it is very important to ensure the balance between supply and demand of natural gas in Chongqing. The first case of COVID‐19 in China was found in Hubei Province, and Hubei Province is also the province most affected by COVID‐19 in China. Chongqing is close to Hubei Province. There are many railways and roads between them, and the personnel flow between the two places is great. At the beginning of COVID‐19 epidemic, Chongqing became the municipality with the largest number of confirmed cases in COVID‐19 due to its geographical location. With the continuous development of the epidemic, the stable supply of natural gas in Chongqing is facing new challenges.

### 4.2 Data preparation

In this paper, we select the quarterly natural gas consumption data of Chongqing from 2011 to 2020 released on the official website of Chongqing Statistics Bureau. However, since the quarterly natural gas consumption data on the official website are quarterly accumulative data, the actual quarterly data in this paper was got by inverse accumulation which can be seen in Table 2.

**Table 2:** Quarterly consumption of natural gas in Chongqing from 2011 to 2020 (unit: billion m^3^).

Index		2011		2012		2013		2014	2015	2016	2017	2018	2019	2020
The first quarter		13.64		15.82		17.36		18.32	18.89	22.60	24.99	23.28	24.82	26.17
The second quarter		14.75		17.08		17.96		21.48	18.46	21.62	23.17	24.41	25.72	25.31
The third quarter		16.03		18.58		17.21		20.10	20.34	21.43	23.08	24.75	26.12	26.74
The fourth quarter		17.22		19.29		19.27		21.73	22.34	23.67	24.00	27.06	26.85	–

*Note*: Data sources: http://data.tjj.cq.gov.cn/tablequery.htm?code=AF07.

**Figure 3:**
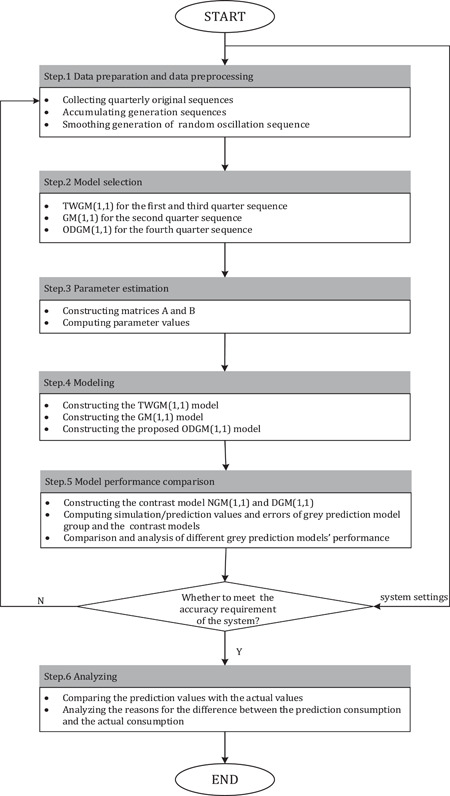
The flowchart for analyzing the impact of COVID‐19 on natural gas consumption in Chongqing.

Considering the data characteristics of Chongqing's quarterly natural gas consumption, we use ODGM(1,1), proposed in this paper, GM(1,1) and TWGM(1,1) to predict the natural gas consumption in 2020, and then analyze the impact of COVID‐19 on natural gas consumption in Chongqing. The flowchart is shown in Figure 3.

### 4.3 New sequences after data preprocessing

The quarterly consumption of natural gas in Chongqing is characterized by an overall increase but random oscillation. The performance of grey prediction models would be poor if we used the original sequences directly. To improve the prediction accuracy, we first use operators to process the quarterly consumption data and then build the models. For the sequences of the first three quarters, they only oscillated randomly. Thus, 1‐AGO will be used to process them before modeling. However, the sequence of the fourth quarter oscillated globally, so we used the smoothing operator and then the 1‐AGO before modeling. According to the statistical data of natural gas consumption in the first three quarters of 2011–2019 in Table 2, the modeling sequences are as follows:

$$X_{1}=(x_{1}\left(1\right),x_{1}\left(2\right),\text{…},x_{1}\left(9\right))=(13.64,15.82,17.36,\;18.32,18.89,22.60,24.99,23.28,24.82),$$

$$X_{2}=(x_{2}\left(1\right),x_{2}\left(2\right),\text{…},x_{2}\left(9\right))=(14.75,17.08,17.96,\;21.48,18.46,21.62,23.17,24.41,25.72),$$

$$X_{3}=(x_{3}\left(1\right),x_{3}\left(2\right),\text{…},x_{3}\left(9\right))=(16.03,18.58,17.21,\;20.1,20.34,21.43,23.08,24.75.26.12).$$

According to Definition 1, the new sequences after 1‐AGO processing are:

$$X_{1}^{\left(1\right)}=\left(x_{1}^{\left(1\right)}\left(1\right),x_{1}^{\left(1\right)}\left(2\right),\text{…},x_{1}^{\left(1\right)}\left(9\right)\right)=(13.64,29.46,46.82,65.14,84.03,106.63,\;131.62,154.9,179.72),$$

$$X_{2}^{\left(1\right)}=\left(x_{2}^{\left(1\right)}\left(1\right),x_{2}^{\left(1\right)}\left(2\right),\text{…},x_{2}^{\left(1\right)}\left(9\right)\right)=(\;14.75,\;31.83,49.79,71.27,89.73,111.35,134.52,\;158.93,184.65),$$

$$X_{3}^{\left(1\right)}=\left(x_{3}^{\left(1\right)}\left(1\right),x_{3}^{\left(1\right)}\left(2\right),\text{…},x_{3}^{\left(1\right)}\left(9\right)\right)=(\;16.03,\;34.61,51.82,71.92,92.26,113.69,136.77,\;161.52,187.64).$$

According to the statistical data of natural gas consumption in the fourth quarter of 2011–2019 in Table 2, the modeling sequence is as follows:

$$X_{4}=(x_{4}\left(1\right),x_{4}\left(2\right),\text{…},x_{4}\left(9\right))=(17.22,\;19.29,19.27,21.73,22.34,23.67,24.00,\;27.06,26.85).$$

According to Definition 5, the new sequence after smooth operator and 1‐AGO is

$$X_{4}^{\left(1\right)}D=\left(x_{4}^{\left(1\right)}\left(3\right)d,x_{4}^{\left(1\right)}\left(4\right)d,\text{…},x_{4}^{\left(1\right)}\left(9\right)d\right)=(\;14.0475,28.6075,43.7775,59.715,\;76.1375,92.975,110.66).$$

### 4.4 Constructing the grey prediction model group of quarterly natural gas consumption in chongqing

According to Theorems 1–4, the related parameters of the grey prediction model group were calculated by MATLAB and the results are shown in Table 3.

**Table 3:** Relevant parameter values of the grey prediction model group.

Index	*a*	*b*	*c*	α	β	γ
$X_{1}$	0.0843	3.1466	12.562	−23.766	37.3377	–
$X_{2}$	−0.0569	15.9232	–	–	–	–
$X_{3}$	−0.14919	−1.9745	17.0682	3.9485	13.2349	–
$X_{4}$	0.0141	0.8309	13.2196	14.5798	0.9860	0.8251

The TWGM(1,1) model for predicting natural gas consumption in the first and third quarters can be constructed by substituting the corresponding parameter values in Table 3 into Equation (6), and the time response equation can be obtained as follows:

(21) $${\hat{x}}_{1}\left(k\right)=\alpha e^{-a\left(k-1\right)}+\beta =-23.766e^{-0.0843(k-1)}+37.3377,$$

(22) $${\hat{x}}_{3}\left(k\right)=\alpha e^{-a\left(k-1\right)}+\beta =3.9485e^{0.14919\left(k-1\right)}+13.2349.$$

The GM(1,1) model for predicting natural gas consumption in the second quarter can be constructed by substituting the corresponding parameter values in Table 3 into Equation (2), and the time response equation can be obtained as follows:

(23) $${\hat{x}}_{2}\left(k\right)=\left(1-e^{a}\right)\left(y_{1}-\frac{b}{a}\right)e^{-a\left(k-1\right)}=\left(1-e^{-0.0569}\right)\;\left(y_{1}-\frac{15.9232}{\left(-0.0569\right)}\right)e^{0.0569\left(k-1\right)},$$

where k=2,3,…. The ODGM(1,1) model for predicting natural gas consumption in the fourth quarter can be constructed by substituting the corresponding parameter values in Table 3 into Equations (11) or (12), and the time response equation can be obtained as follows:

(24) $${\hat{x}}_{4}\left(k+1\right)=\frac{4\alpha \left(\beta^{k-1}-1\right)}{1+\beta }+\frac{4\gamma \left(1-\beta^{k-1}\right)}{1-\beta^{2}}+{\left(-1\right)}^{k+1}x\left(2\right)-\left(1+{\left(-1\right)}^{k}\right)T=\frac{4\times 14.5798\left(0.98596^{k-1}\right)}{1+0.98596}+\frac{4\times 0.8251\left(1-0.98596^{k-1}\right)}{1-0.98596^{2}}+{\left(-1\right)}^{k+1}x\left(2\right)-\left(1+{\left(-1\right)}^{k}\right)9.84,$$

where k−2,3,….

### 4.5 Comparing the performance of grey prediction model group and other traditional grey prediction models

To compare the simulation and prediction performances of the grey prediction model group, NGM(1,1) and DGM(1,1) were also applied to simulate and predict the quarterly nature gas consumption in Chongqing. All quarterly simulation and prediction results of the above models for different quarters are shown in Tables 4, 5, 6, 7 respectively. The parameters of models in the grey prediction model group are shown in Table 3, so their parameters are not shown in Tables 4, 5, 6, 7.

**Table 4:** Comparisons of simulation/prediction errors of the three models for the sequence $X_{1}$

Year	$x^{\left(0\right)}\left(k\right)$	TWGM(1,1)		NGM(1,1,*k*) *a* = 0.4645; *b* = 11.2913		DGM(1,1) *a* = 1.0673; *b* = 15.4403	
		${\hat{x}}_{1}\left(k\right)$	$\Delta_{k}\left(k\right)$	${\hat{x}}_{1}\left(k\right)$	$\Delta_{k}$	${\hat{x}}_{1}\left(k\right)$	$\Delta_{k}\left(k\right)$
2011	13.64	–	–	–	–	–	–
2012	15.82	15.4925	2.0702	8.8284	44.1946	16.3584	3.4034
2013	17.36	17.2580	0.5873	14.5802	16.0126	17.4595	0.5734
2014	18.32	18.8809	3.0615	18.1950	0.6823	18.6348	1.7183
2015	18.89	20.3726	7.8484	20.4668	8.3471	19.8892	5.2894
2016	22.60	21.7437	3.7890	21.8945	3.1218	21.2280	6.0710
2017	24.99	23.0040	7.9472	22.7917	8.7965	22.6569	9.3363
2018	23.28	24.1625	3.7906	23.3556	0.3249	24.1820	3.8744
2019	24.82	25.2273	1.6409	23.7100	4.4721	25.8097	3.9876
2020	26.17	26.2060	0.1376	23.9328	8.5487	27.5470	5.2618
$\bar{\Delta }$			3.8419		10.7440		4.2817

**Table 5:** Comparisons of simulation/prediction errors of the three models for the sequence $X_{2}$

Year	$x^{\left(0\right)}\left(k\right)$	GM(1,1)		NGM(1,1,*k*) *a* = 0.5770; *b* = 13.6218		DGM(1,1) *a* = 1.0583; *b* = 16.4107	
		${\hat{x}}_{1}\left(k\right)$	$\Delta_{k}\left(k\right)$	${\hat{x}}_{1}\left(k\right)$	$\Delta_{k}$	${\hat{x}}_{1}\left(k\right)$	$\Delta_{k}\left(k\right)$
2011	14.75	14.75	–	–	–	–	–
2012	17.08	17.24	0.9874	9.5529	44.0694	17.271	1.1172
2013	17.96	18.2586	1.6628	15.7145	12.5026	18.278	1.7704
2014	21.48	19.3278	10.0198	19.1746	10.7327	19.344	9.9448
2015	18.46	20.4595	10.8315	21.1177	14.3968	20.472	10.899
2016	21.62	21.6575	0.1734	22.2088	2.7232	21.666	0.2115
2017	23.17	22.9256	1.0547	22.8215	1.5041	22.929	1.0394
2018	24.41	24.2680	0.5816	23.1656	5.0980	24.266	0.5887
2019	25.72	25.6890	0.1204	23.3588	9.1804	25.681	0.1501
2020	25.31	27.1933	7.4408	23.4673	7.2805	27.179	7.38
$\bar{\Delta }$			3.1789		12.5259		3.2151

**Table 6:** Comparisons of simulation/prediction errors of the three models for the sequence $X_{3}$

Year	$x^{\left(0\right)}\left(k\right)$	TWGM(1,1)		NGM(1,1,*k*) *a* = 0.6042; *b* = 14.3445		DGM(1,1) *a* = 1.0582; *b* = 16.5174	
		${\hat{x}}_{1}\left(k\right)$	$\Delta_{k}\left(k\right)$	${\hat{x}}_{1}\left(k\right)$	$\Delta_{k}$	${\hat{x}}_{1}\left(k\right)$	$\Delta_{k}\left(k\right)$
2011	16.03	–	–	–	–	–	–
2012	18.58	17.8187	4.0976	9.4188	49.3070	17.45	6.0831
2013	17.21	18.5562	7.8221	15.9137	7.5322	18.465	7.2905
2014	20.1	19.4123	3.4212	19.4632	3.1681	19.539	2.7927
2015	20.34	20.4062	0.3257	21.4030	5.2262	20.675	1.6475
2016	21.43	21.5601	0.6070	22.4631	4.8209	21.878	2.0888
2017	23.08	22.8995	0.7819	23.0425	0.1626	23.15	0.3037
2018	24.75	24.4545	1.1938	23.3591	5.6199	24.497	1.0239
2019	26.12	26.2597	0.5348	23.5321	9.9077	25.921	0.7604
2020	26.74	28.3553	6.0408	23.6267	11.6429	27.429	2.58
$\bar{\Delta }$			2.348		10.7181		2.7488

**Table 7:** Comparisons of simulation/prediction errors of the three models for the sequence ESE31164

Year	$x^{\left(0\right)}\left(k\right)$	ODGM(1,1)		NGM(1,1,*k*) *a* = 0.7191; *b* = 17.5411		DGM(1,1) *a* = 1.05; *b* = 18.2991	
		${\hat{x}}_{1}\left(k\right)$	$\Delta_{k}\left(k\right)$	${\hat{x}}_{1}\left(k\right)$	$\Delta_{k}$	${\hat{x}}_{1}\left(k\right)$	$\Delta_{k}\left(k\right)$
2011	17.22	17.22	–	–	–	–	–
2012	19.29	19.29	–	10.6759	44.6558	19.1595	0.6763
2013	19.27	19.3491	0.4107	17.7100	8.0953	20.1168	4.3946
2014	21.73	21.7716	0.1916	21.1369	2.7293	21.1220	2.7980
2015	22.34	21.7959	2.4353	22.8065	2.0880	22.1773	0.7281
2016	23.67	24.1841	2.1719	23.6199	0.2120	23.2854	1.6247
2017	24.00	24.1745	0.7272	24.0161	0.0670	24.4489	1.8704
2018	27.06	26.5293	1.9612	24.2091	10.5354	25.6705	5.1349
2019	26.80	26.4868	1.3527	24.3032	9.4854	26.9531	0.3841
2020	–	28.8091	–	24.3490	–	28.2998	–
$\bar{\Delta }$			1.3215		9.7335		2.2014

The predicted data of four quarters of 2020 will be compared with the actual data after the outbreak of COVID‐19. Thus, the relative percentage errors (RPEs) of the predicted data in 2020 are not included in the average RPE calculation. The RPE$\Delta_{k}$ and the mean RPE (MRPE)$\bar{\Delta }$ are shown in Table 4 are as follows:

$$\Delta_{k}=\frac{|{\hat{x}}_{i}\left(k\right)-x_{i}^{\left(0\right)}\left(k\right)|}{x_{i}^{\left(0\right)}\left(k\right)}\times 100\%;\;\bar{\Delta }=\frac{\sum_{k=2}^{n}\Delta \left(k\right)}{n-1}\times 100\%.$$

**Figure 4:**
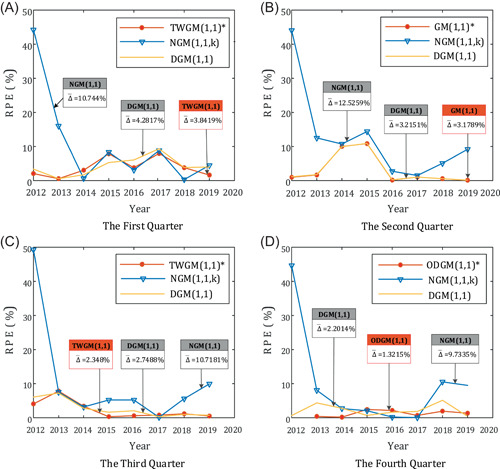
The line chart of relative percentage error (RPE) in the four quarters

**Figure 5:**
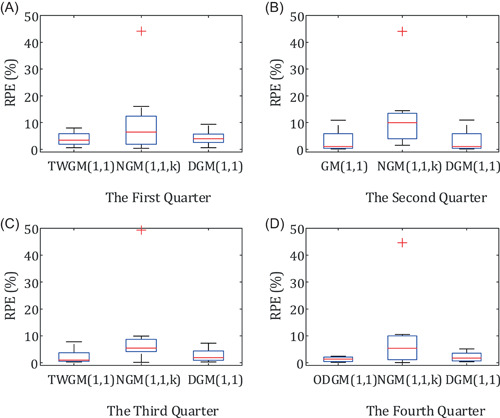
The box chart of relative percentage error (RPE) of different models in the four quarters

To visually compare the difference between the grey prediction model group and other models in fitting effect, the relative percentage errors diagrams of different grey models in different quarters are drawn and shown in Figures 4 and 5. The line chart is to intuitively compare the fitting effect of the grey prediction model group and other grey models, and the box chart is to intuitively show the distribution of RPE of each model in different quarters.

According to Tables 4, 5, 6, 7, some conclusions can be given as follows:

1. From the overall respective, the simulation and prediction performances of the models in grey prediction model group are better than the NGM(1,1) model and the DGM(1,1) model.
2. The MRPE of grey prediction model group and the DGM(1,1) model are lower than 5%, indicating precision grades of those grey models are between Classes Ι and II and the fitting effect of those grey models are good. All of the MRPE of the NGM(1,1) model in four quarters are higher than 10%, and the fitting effect of the NGM(1,1) model is worst.
3. For the sequence $X_{4}$ with global oscillation, the simulation and prediction effect of the ODGM(1,1) model proposed in this paper is better than other traditional prediction models.
4. For the sequence $X_{2}$, the MRPE of the GM(1,1) model is close to the DGM(1,1) model, but the GM(1,1) model is slightly better than the DGM(1,1) model.

### 4.6 Comparative analysis

As is presented in Tables 4, 5, 6, 7, the predicted consumption of natural gas for the first three quarters in 2020 are 26.2060 × 10^8^, 27.1933 × 10^8^, and 28.3553 × 10^8^ m^3^, respectively. Compared with the actual consumption, the predicted consumption in the first three quarters of 2020 decreases by 0.1376%, 7.4408%, and 6.0408%, respectively. It shows that COVID‐19's impact on Chongqing's natural gas consumption in the first quarter of 2020 is much less than in the second and third quarters. The reasons why COVID‐19's impact on Chongqing's natural gas consumption in the first quarter of 2020 is tiny are as follows:

1. During the traditional Chinese spring festival, household natural gas consumption accounts for a relatively larger proportion compared with other time of the year. Besides, COVID‐19 broke out in late January 2020, coinciding with the spring festival and the home quarantine policy made people stay at home. COVID‐19 had little negative impact on household natural gas consumption.
2. Chongqing is an industrial city. To ensure the stable supply of medical and living necessities during the epidemic, many enterprises in Chongqing started production ahead of schedule. Some medical supplies production enterprises were even in a 24‐h nonstop state during the Spring Festival. It was the extra consumption of natural gas in such special enterprises that increased the total amount of consumption in the first quarter, weakening the impact of COVID‐19 on the surface.
3. In late February and early March, Chongqing's government had taken a series of measures to actively promote the resumption of work and production of enterprises. These factors have reduced the impact of the epidemic on industrial natural gas consumption to a certain extent.

With the gradual control of the domestic epidemic in China, the medical and living supplies became gradually stable. The special case in which some specific enterprises consumed a large amount of natural gas in the early stage of the epidemic had disappeared. However, most industries in Chongqing have been going through a difficult time since it needs time for the society to recover completely. What's more, the spread of the epidemic in foreign countries makes imports and exports more difficult than ever before. According to the official website of Chongqing Statistics Bureau, the cumulative added values of Chongqing's GDP in the second and third quarters of 2020 were 0.8% and 2.6%, respectively, being the lowest compared with the same period in the past decade. It shows that the sudden occurrence of COVID‐19 had a certain impact on the social life and economic development of Chongqing, which affected the consumption of natural gas indirectly and prominently in the second and third quarters of 2020. However, COVID‐19's impact on natural gas consumption in the third quarter is less than that in the second quarter, indicating that the society has been gradually backing to normal.

## 5 CONCLUSION

A novel grey prediction model based on smooth operator, ODGM(1,1) for short, was proposed in this paper. It improved the simulation and prediction accuracy of overall oscillation sequences. Then, the grey prediction group, consisting of ODGM(1,1), TWGM(1,1), and GM(1,1) was employed to predict Chongqing's natural gas consumption in the absence of COVID‐19 in 2020. Finally, we compared the predicted consumption values with the actual consumption values and found that COVID‐19 had a slight impact on the consumption of natural gas in the first quarter of 2020 and comparatively bigger impact in the second and third quarters. However, as the economy recovered and grew steadily in the fourth quarter of 2020, we predicted that the natural gas consumption of Chongqing in the fourth quarter would be close to the consumption predicted by ODGM(1,1). To sum up, in the early days of COVID‐19, the increase in household gas consumption and the abnormal production of some special enterprises had led to an increase in natural gas consumption in Chongqing. Thus, it seemed that the natural gas consumption in the first quarter was less affected by the epidemic. However, the short‐term interference did not affect the medium and long‐term trend. With the continuous spread of the epidemic, its impact on natural gas consumption would gradually become prominent. After the government took some measures to reduce the impact of the incident on the social economy and people's daily life, the natural gas consumption would gradually return to the normal state of growth.

## CONFLICTS OF INTEREST

The authors declare no conflicts of interest.
